# Treatment efficiency and quality improvement via double imaging modality (DIM) versus single imaging modality (SIM) image-guided radiotherapy for prostate cancer

**DOI:** 10.1016/j.tipsro.2025.100307

**Published:** 2025-02-21

**Authors:** Milad Mirzaei, Suki Gill, Mahsheed Sabet, Martin A. Ebert, Pejman Rowshanfarzad, Jake Kendrick, Angela Jacques, Clare Herbert, Jeremy Croker, Sean Bydder, Joshua Dass, Edward Bailey, Rohen White, Catherine Moffat, Colin Tang, Adriano Polpo, Nicholas Bucknell

**Affiliations:** a Department of Radiation Oncology, Sir Charles Gairdner Hospital, Nedlands, WA, Australia; b School of Physics, Mathematics and Computing, University of Western Australia, Crawley, WA, Australia; c Centre for Advanced Technologies in Cancer Research, Perth, WA, Australia; d Institute for Health Research, University of Notre Dame, Fremantle, WA, Australia; e Edith Cowan University, Joondalup, WA, Australia; f Sir Peter MacCallum Department of Oncology, University of Melbourne, Melbourne, VIC, Australia

**Keywords:** IGRT, Prostate, Anteroposterior kilovoltage imaging (AP-kV), CBCT, Bladder, Rectal gas

## Abstract

**Purpose:**

Daily image-guided radiotherapy (IGRT) for prostate cancer reduces treatment-related toxicity. However, positional and anatomical variations can result in patient re-setup, increased treatment time, and additional imaging dose. A simple technique of two distinct imaging modalities was investigated: initially, an anteroposterior kilovoltage (AP-kV) planar image was acquired, followed by cone beam computed tomography (CBCT). To determine whether this dual imaging modality (DIM) can reduce repeat CBCTs with an AP-kV screening image compared to CBCT alone, *i.e.* a single imaging modality (SIM).

**Methods:**

Patients were enrolled sequentially before and after the new departmental protocol for IGRT. Initially, all patients had a CBCT prior to treatment (SIM group) as usual. For the DIM group, AP-kV imaging was added to the first three fractions, and subsequent AP-kV imaging (on demand) for patients unable to maintain adequate bladder and bowel compliance. Sixty intact prostate or post-prostatectomy patients were included: 30 before the change in protocol (SIM group) and 30 after (DIM group). Bladder volume, rectal filling, and positioning errors were evaluated.

**Results:**

In the SIM group, 30 patients underwent a total of 1116 CBCTs. In the DIM group, 30 patients had 156 AP-kV images and 1077 CBCTs. In the SIM group, 166 were repeat CBCTs with a median of 4 repeat CBCTs per patient. In DIM group, 132 were repeat CBCTs with a median of 3 repeat CBCTs per patient. We found a significant difference in incidence of repeat CBCTs due to suboptimal bladder filling (p = 0.028) and rectal gas (p = 0.045), indicating the number of repeat CBCTs was significantly lower in patients imaged with DIM.

**Conclusion:**

The DIM technique was found to be superior to the SIM technique, as it allowed the desired bladder volume, rectal condition, and patient positioning to be achieved prior to CBCT, reducing the need for repeat CBCTs.

## Highlights

• A low dose AP-kV image can serve as a rapid bladder and rectal gas screening procedure for prostate radiotherapy.
• Daily AP-kV imaging prior to CBCT can reduce the number repeat CBCTs in prostate patients undergoing radiotherapy.
• Double imaging modality (DIM) for prostate patients improves efficiency in busy radiotherapy departments.

## Introduction

Image-guided radiotherapy (IGRT) is the standard of care in radiotherapy (RT) for prostate cancer and has proven effective in reducing both acute and late radiation-induced toxicities [1], [2]. Rectal filling status emerges as the most significant predictor of intra-fractional prostate motion, where increased rectal size prior to treatment can lead to greater prostate motion during treatment and an increased likelihood of geographic miss [3]. Maintaining a comfortably full bladder and an empty rectum during RT significantly improves treatment accuracy and minimises the risk of radiation-induced toxicities in prostate cancer patients [4], [5]. A less-full bladder may fall into the treated volume during treatment, potentially resulting in a larger portion of the bladder being exposed to undesired high doses. Meanwhile, a large rectum could either tilt the prostate anteriorly or cause organ deformation, both of which may lead to geographic miss.

Currently, our department offers two IGRT modalities for prostate RT, orthogonal kilovoltage (kV) planar imaging (kV PI), and kV cone-beam computed tomography (CBCT). kV PI is primarily acquired in the anteroposterior (AP) and lateral directions. They provide high contrast two-dimensional images that allow Radiation Therapy Technologists (RTTs) to correct positional errors based on patient bony anatomy and fiducial marker position. In contrast, CBCT image sets provide three-dimensional volumetric scans with either 200° (Spotlight) or 360° (Full) gantry rotation. Considerable research has been conducted comparing the advantages and disadvantages of these IGRT modalities for prostate patients in terms of radiation exposure, efficiency, accuracy, and precision [6], [7], [8]. In 2016, a National Survey of American Society for Radiation Oncology Members showed that 92% used volumetric imaging with CBCT for IGRT [9]. kV PI of fiducial markers allows for the correction of setup errors and prostate displacement, while CBCT enables RTTs to verify that the bladder is adequately filled and the rectum is properly emptied before treatment. If these conditions are not met, the patient is sometimes removed from the bed and coached to either drink more water, empty the rectum, or both, depending on institutional protocols.

Monte Carlo simulations has shown that kV PI leads to lower radiation dose exposure and faster image acquisition than CBCT in Varian on-board imaging systems (Varian Medical Systems, Palo Alto, CA) [10]. Radiation dose will be higher if the patient needs repositioning after the initial CBCT, and a repeat (verification) CBCT is acquired following repositioning. Furthermore, daily variations in bladder and rectum conditions can lead to increased treatment time, random errors, and workflow inefficiencies, particularly in busy departments that do not use an ultrasound bladder scanner before treatment fractions to assess bladder volume. Such prolonged procedures could also cause frustration and psychological distress, particularly in elderly patients [11].

This study evaluates a simple solution using dual imaging modalities (DIM) for prostate IGRT, which was implemented in our busy department to minimise the number of repeat CBCTs and patient delays. In this approach, very low-dose AP-kV imaging serves as an initial screening procedure prior to CBCT. The purpose of this study was to assess the efficacy of AP-kV imaging and analyse whether the DIM technique could reduce the number of repeat CBCTs compared to the single imaging modality (SIM) technique, which relies solely on CBCT.

## Methods

This study was approved as a quality improvement project by the hospital ethics board (Quality Activity 52132).

### Patients

All patients included in this study had histologically confirmed prostate adenocarcinoma and were suitable candidates for either intact prostate or post-prostatectomy IGRT. All patients underwent CT simulation and RT in a head-first supine position, stabilised with a medium-sized pillow under head, a Combifix™ with adjustable Kneefix™ and Feetfix™ patient support cushions. Before simulation and each treatment fraction, patients were instructed to empty their bowels, and drink between 200–1000 ml of water 30–70 minutes prior to treatment. This was adjusted based on bladder filling during treatment, with RTTs instructing patients to drink more or wait longer depending on bladder size.

All patients received treatment on Varian TrueBeam linear accelerators (Varian Medical Systems, Palo Alto, CA). Pre-treatment images were obtained using the kV imaging system (On-Board Imager®).

Patients were enrolled sequentially before and after the implementation of a new departmental protocol for IGRT. Initially, all patients had a single CBCT prior to treatment (SIM). Our department then added AP-kV imaging for the first three fractions and subsequent AP-kV imaging (on demand) for patients unable to maintain adequate bladder and bowel compliance.

### Image acquisition, evaluation, and processing

#### SIM

For the SIM patient group, daily CBCT imaging was performed on all patients. The CBCT acquisition parameters were set as follows: Pelvis, half fan bow tie filter, 125 kV, 1056 mAs, and 360° gantry rotation. CBCTs were scheduled with a gantry start angle of 180.0° (counterclockwise).

During the image evaluation process, RTTs performed an automatic bone match to the pelvic bones, followed by a fiducial marker match (if fiducials were present). Subsequently, RTTs evaluated conditions of the target volumes and critical structures, including the bladder and rectum. As per our departmental protocol, patient repositioning and repeat CBCT was required if the bladder volume was not within the acceptable range of approx. 80–120%, if the rectum was full of faeces compromising target coverage, if a large rectal gas bubble (>3.0 cm in diameter) was present, or in the event of a large pelvic shift (>1.0 cm translationally or >1.5° rotationally).

#### DIM

**Fig. 1:**
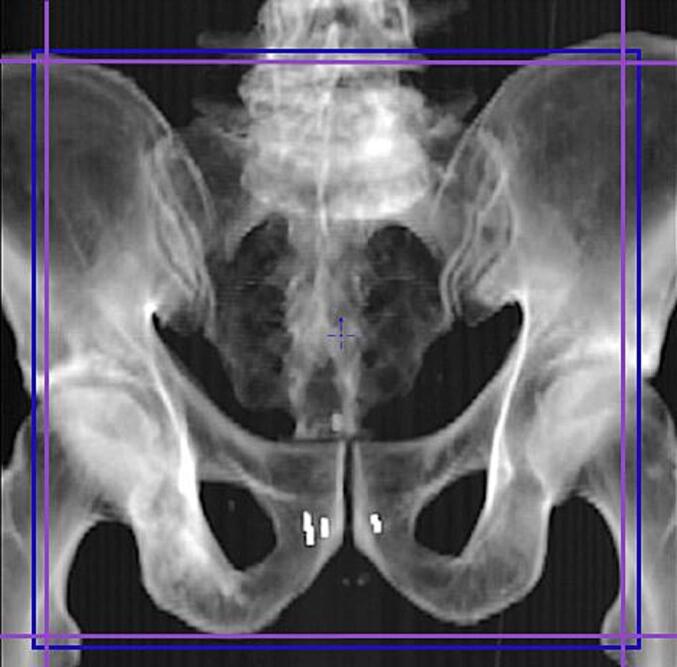
Illustration of the appropriate field size and imager position for AP-kV imaging of the pelvis on a DRR. Prior to imaging, the kV imager position (blue rectangle) and the kV imaging blades (purple lines) were adjusted by selecting the Collimation and Blades Tracking tools. (For interpretation of the references to colour in this figure legend, the reader is referred to the web version of this article.)

For the DIM patient group, the AP-kV field size (approx. 20 x 20 cm) encompassed the uppermost part of the bilateral ilium superiorly, the inferior ischial rami inferiorly, and the bilateral femoral heads laterally (Fig. 1). RTTs were instructed to select the recommended kV acquisition parameters and settings for the AP-kV imaging. A single AP-kV image was acquired with the parameters set to the default Pelvis Small protocol (75 kV, 3 mAs).

**Fig. 2:**
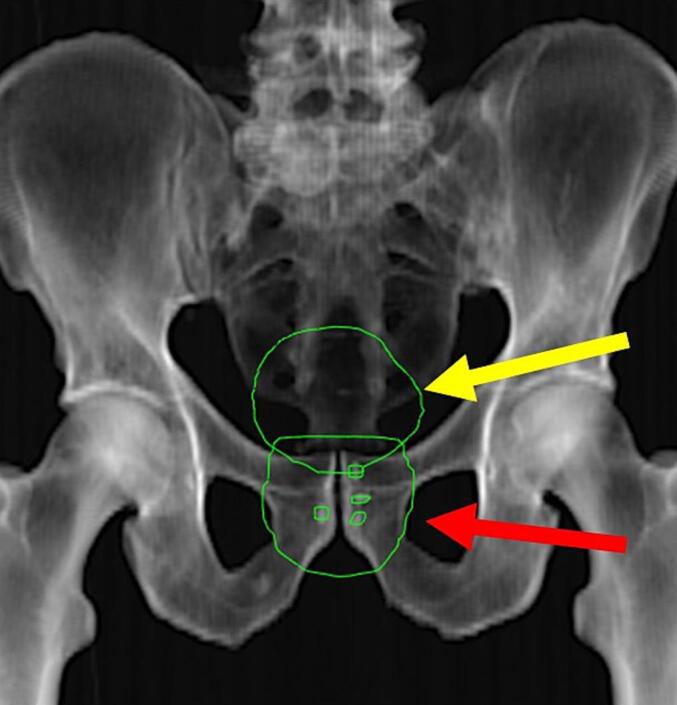
Representation of the AP-kV DRR of a prostate patient. The yellow arrow points to the bladder contour, while the red arrow shows the PTV, which encompasses the four fiducial marker contours. (For interpretation of the references to colour in this figure legend, the reader is referred to the web version of this article.)

During the image evaluation process, RTTs attached the planned bladder, planning target volume (PTV), and fiducial marker contours to the AP-kV digitally reconstructed radiographs (DRR) for each patient (Fig. 2).

**Fig. 3:**
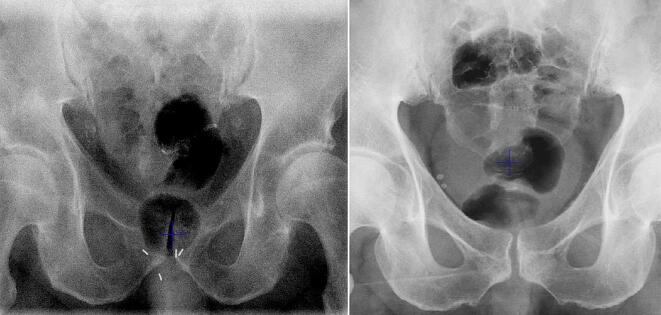
AP-kV images of two prostate patients exposing the bladder shadow, rectal gas bubbles, and the bony anatomy. In patient 1 (Left image), the AP-kV revealed a clinically relevant rectal gas posterior to the fiducial markers. As a result, RTTs decided to remove the patient from the bed before proceeding to CBCT. In patient 2 (Right image), the AP-kV exposed both the bladder and bowel gas, but RTTs decided to proceed to CBCT as the gas was located superiorly and far from the PTV.

Each RTT independently attempted to visualise the bladder and rectal gas in each AP-kV image. The Varian TrueBeam image display filters (*i.e.* Content, Highlight, Brightness) were manually adjusted to optimise clarity. After image matching using the appropriate procedure (*i.e.* setting up to the fiducial markers inside the prostate for intact prostate IGRT or setting up to pelvic bones for post-prostatectomy IGRT), RTTs visually compared the exposed bladder shadow to the planned bladder contour and approximated the volume (*e.g.* bladder <50% or bladder 100%). As per our departmental protocols, patients were removed from the bed before proceeding to CBCT, and another AP-kV imaging attempt was made later if the exposed bladder volume was not within the acceptable range of approx. 80–120% and/or a large rectal gas bubble (>3.0 cm in diameter) was detected (Fig. 3) or if repositioning of the patient was needed due to a large pelvic shift.

Repeat CBCTs were categorised into five groups with reference to the planning CT. The groups were as follows: TOB-PI (patient was taken off the bed − due to positioning issues), TOB-FG (patient was taken off the bed − due to faeces and gas), TOB-BS (patient was taken off the bed − due to small bladder volume defined as <50% compared to the planning CT), TOB-RGBS (patient was taken off the bed − due to rectal gas and small bladder volume <50%), TOB-BXL (patient was taken off the bed − due to large bladder volume >120%) (Table 2).

**Table 1:** Patient demographics and clinical data.

	SIM	DIM
	(N = 30)	(N = 30)
Age (y)		
Median (Q1, Q3)	68 (59, 74)	72.5 (68, 80)
Min, Max	51, 83	59, 89
Bladder filling instruction (ml)		
Median (Q1, Q3)	600 (600, 600)	600 (600, 600)
Min, Max	200, 850	400, 1000
Bladder filling wait time (min)		
Median (Q1, Q3)	40 (30, 50)	45 (40, 45)
Min, Max	30, 70	30, 60
Radiation dose (Gy)		
Median (Q1, Q3)	66 (64, 67)	66 (64, 78)
Min, Max	55, 78	60, 78
Fraction (Fx)		
Median (Q1, Q3)	33 (32, 35)	33 (25, 39)
Min, Max	20, 39	3, 39
Total CBCTs per patient (n)		
Median (Q1, Q3)	38 (34, 42)	36 (32, 43)
Min, Max	20, 55	20, 50
Accepted CBCTs (n)		
Median (Q1, Q3)	33 (32, 35)	33 (25, 39)
Min, Max	20, 39	20, 39
Repeat CBCTs (n)		
Median (Q1, Q3)	4 (2, 8)	3 (1, 7)
Min, Max	0, 16	0, 15
Accepted CBCTs % (of total CBCT)		
Median (Q1, Q3)	89.2 (80.9, 91.7)	90.9 (83.0, 97.2)
Min, Max	67.3, 100.0	69.4, 100.0
Repeat CBCTs % (of total CBCT)		
Median (Q1, Q3)	10.8 (8.3, 19.1)	9.1 (2.8, 17.0)
Min, Max	0.0, 32.7	0.0, 30.6

**Table 2:** Patient CBCT classification and reasons for removing patients from the bed.

	SIM	DIM
	(N = 30)	(N = 30)
	n (%)	n (%)
Taken off the bed – positioning issues (TOB-PI)		
Any	14 (46.7 %)	9 (30.0 %)
# times		
*Median (Q1, Q3)*	0 (0, 1)	0 (0, 1)
*Min, Max*	0, 5	0, 4
# times category		
0	16 (53.3 %)	21 (70.0 %)
1	9 (30.0 %)	6 (20.0 %)
>1	5 (16.7 %)	3 (10.0 %)
Taken off the bed – rectal gas (TOB-RG)		
Any	15 (50.0 %)	20 (66.7 %)
# times		
*Median (Q1, Q3)*	1 (0, 1)	1 (0, 3)
*Min, Max*	0, 13	0, 9
# times category		
0	15 (50.0 %)	10 (33.3 %)
1	8 (26.7 %)	6 (20.0 %)
>1	7 (23.3 %)	14 (46.7 %)
Taken off the bed – rectal gas and bladder volume < 50% (TOB-RGBS)		
Any	12 (40.0 %)	5 (16.7 %)
# times		
*Median (Q1, Q3)*	0 (0, 1)	0 (0, 0)
*Min, Max*	0, 3	0, 2
# times category		
0	18 (60.0 %)	25 (83.3 %)
1	8 (26.7 %)	4 (13.3 %)
>1	4 (13.3 %)	1 (3.3 %)
Taken off the bed – bladder volume < 50% (TOB-BS)		
Any	24 (80.0 %)	16 (53.3 %)
# times		
*Median (Q1, Q3)*	2 (1, 3)	1 (0, 2)
*Min, Max*	0, 11	0, 5
# times category		
0	6 (20.0 %)	14 (46.7 %)
1	8 (26.7 %)	5 (16.7 %)
>1	16 (53.3 %)	11 (36.7 %)
Taken off the bed – bladder volume > 120% (TOB-BXL)		
Any	4 (13.3 %)	3 (10.0 %)
# times		
*Median (Q1, Q3)*	0 (0, 0)	0 (0, 0)
*Min, Max*	0, 2	0, 2
# times category		
0	26 (86.7 %)	27 (90.0 %)
1	3 (10.0 %)	1 (3.3 %)
>1	1 (3.3 %)	2 (6.7 %)

### Statistical analysis

Continuous data are summarised as median and interquartile range with Mann-Whitney U tests used for group comparisons. Categorical data are summarised as frequency distributions and compared using Chi squared tests. Stata version 18 (StataCorp, College Station, TX) was used for data analysis and p-values <0.05 were considered statistically significant.

## Results

Table 1 summarises the patient demographics and clinical information. For the entire group, the median volume of water drunk by patients was 600 ml, and the median time patients were advised to start drinking before treatment was 42.5 min. The prescribed dose ranged from 55 Gy in 20 fractions to 78 Gy in 39 fractions (median 66 Gy in 33 fractions), depending on high-risk or intermediate-risk prostate cancer, and whether the treatment was radical, palliative, definitive, or postoperative.

### SIM

The SIM patient group included 1116 CBCTs from thirty prostate patients, aged 51–83 years (median 68 years), treated with RT between May and October 2021 at a single tertiary hospital. 11 patients required treatment to the prostate bed; 6 to the prostate bed and pelvic nodes; 5 to the prostate; 5 to the prostate and seminal vesicles (SVs); and 3 to the prostate and pelvic nodes. Patients received between 20–39 fractions of RT, with a median of 33. Of the 30 SIM patients, only one had no repeat CBCT. 166 out of 1116 CBCTs were repeated across the other 29 patients. In the SIM group, the median number of repeat CBCTs was 4 (interquartile range 2–8). The most common reasons for repeated imaging was insufficient bladder volume, followed by rectal gas, and then patient positioning issues.

### DIM

The DIM patient group included 156 AP-kV imaging (acquired fractions 1–3 and on demand when deemed necessary by RTTs), in addition to 1077 CBCTs, from thirty prostate patients aged 59 to 89 (median 72.5 years), treated with RT between May and October 2022 at the same hospital. Similar to the SIM group, 11 patients required treatment to the prostate bed; 6 to the prostate bed and pelvic nodes; 5 to the prostate; 5 to the prostate and SVs; and 3 to the prostate and pelvic nodes. Patients received between 20 and 39 fractions of RT, with a median of 33. Of the 30 DIM patients, 6 had no repeat CBCT. A total of 132 out of 1077 CBCTs were repeated across the other 24 patients. In the DIM group, the median number of repeat CBCTs per patient was 3 (interquartile range 1–7). The most common reasons for repeated imaging was insufficient bladder volume, followed by rectal gas, and then patient positioning issues.

Of the 30 DIM patients, all had AP-kV imaging fractions 1 to 3 and on demand. In total, 156 AP-kV were acquired. The average number of AP-kV imaging per patient was 5 (range 3–14). 10 out of 30 patients (over a total of 14 fractions) were removed from the bed after the AP-kV imaging due to insufficient bladder volume, rectal gas, or positioning issues. Following repositioning, confirmation AP-kV images were acquired in 5 patients (over a total 8 fractions) before proceeding to CBCT. No patients required further repositioning after CBCT imaging. In 5 patients (over a total of 6 fractions), RTTs proceeded to CBCT immediately after the AP-kV screening (without removing the patient from the bed), resulting in the need for 6 repeat CBCTs. These repeats were attributed to RTTs errors in selecting specific imaging parameters (as described in the Methods Section), experience and lack of confidence in adequately detecting the faint bladder shadow (2 fractions) and small rectal gas bubbles mixed with faeces (4 fractions) during image evaluation.

**Fig. 4:**
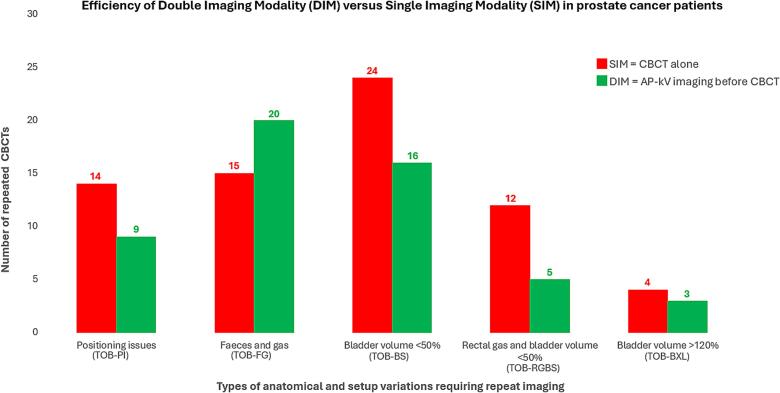
Comparision of the efficiency between DIM and SIM.

This study found a significant difference in incidence of repeat CBCTs in the TOB-BS (p = 0.028) and TOB-RGBS (p = 0.045) groups, indicating that the number of repeat scans was significantly lower in patients imaged with the DIM technique (Fig. 4).

In the TOB-PI group, the DIM technique resulted in fewer repeat CBCTs compared to the SIM technique, but the difference was not statistically significant (p = 0.184). No significant difference was found in the TOB-BXL (p = 0.688) and TOB-FG (p = 0.190) groups.

## Discussion

International committees have mandated the implementation of ‘As Low As Reasonably Achievable’ (ALARA) principle for radiation dose during medical imaging procedures [12], [13]. Various IGRT techniques have been explored to minimise the imaging dose to organs at risk in cancer patients [10], [14], [15], [16]. Standard CBCT imaging can deliver a dose of 1.0–3.96 cGy to the organs, in addition to the prescribed dose during treatment [10]. More specifically, a conventional prostate patient planned for 39 fractions with daily CBCT would receive an additional 1.19–1.79 cGy to the prostate gland per CBCT, depending on the patient's size [10]. Notably, this value would increase with the number of repeated imaging procedures [17]. The present study investigated whether repeated imaging can be reduced by performing daily low-dose AP-kV imaging for the first three fractions and on demand prior to CBCT.

There is no doubt that alternative on-board imaging modalities, such as the Varian Pelvis Spotlight imaging and CBCT with lowered parameters, deliver less concomitant dose compared to standard CBCT over a 39-fraction prostate treatment [10], [14], [15]. However, kV PI has been found to reduce the imaging dose and shorten the time required for image acquisition [10], [18], [19].

The ‘single’ AP-kV imaging technique introduced in this study would still deliver the lowest dose level according to imaging dose studies conducted on these modalities [10], [14]. Additionally, single AP-kV appears to be the shortest in duration (<5 s) compared to the 200° Pelvis Spotlight (approx. 40 s) and Full Pelvis CBCT (approx. 70 s) in Varian on-board imaging systems [20].

Pang *et al.* investigated intra-fractional motion in 60 prostate patients and found a significant reduction in the mean intra-fractional motion in both Sup/Inf (p = 0.008) and Ant/Post (p = 0.0001) directions when the bladder volume was between 82–113%, regardless of the bladder volume in the planning CT [21]. Pearson *et al.* suggested that treating prostate patients with a bladder volume smaller than 75% can increase the cumulative bladder dose V70Gy from 9.5% on the planning CT to 11% during treatment [22]. Chen *et al.* found that a 10% increase in the bladder volume would reduce the mean bladder dose by 5.6% [23]. Gurjar *et al.* highlighted that variations in bladder and rectum volumes can lead to significant prostate positional shift, as well as changes in the delivered dose to the bladder and rectum, and dose coverage of 95% and 93% of the PTV [24].

These findings highlight the importance of performing a prior bladder assessment and allowing more time for the patient’s bladder to reach the optimal volume before RT. In the present study, bladder filling on CBCT was found to be more reproducible in the DIM patient group, or at least in patients whose bladder could be easily visualised by AP-kV imaging, assuming there were no random RTT errors in selecting imaging parameters, such as ensuring an adequate field size as specified in the Methods section.

Several studies have examined the impact of rectal gas on dose distribution during volumetric-modulated arc prostate RT [25], [26], [27], [28]. A significant clinical target volume and PTV dose reduction could result from the presence of ≥5% rectal gas compared to the treatment plan [25]. Oates *et al.* study on image-guided adaptive prostate RT, using rectal diameter as a predictor of motion, demonstrated with 90% confidence that an increased mean rectal diameter of >3.0 cm leads to prostate displacement of >5 mm [29]. A comprehensive study by Wada *et al.* on the effectiveness of rectal gas removal in improving dose distribution and reducing prostate motion during RT found that rectal gas removal significantly improved all dosimetric parameters [26]. In our study, the DIM technique detected and resolved most rectal gas issues prior to the acquisition of the pre-treatment CBCT, or at least in patients in whom the AP-kV could detect large rectal gas bubbles of clinical relevance, assuming there were no random RTT errors. For example, in the TOB-FG group, AP-kV could not visualise faeces mixed with small gas bubbles.

Although the findings of our study do not directly support the implementation of the DIM technique in proton therapy, we suggest that it may have potential utility as a screening tool in prostate image-guided proton therapy. This proposition warrants further investigation and presents a compelling avenue for future research.

The primary limitation of the present study lies in the categorisation of the reasons for repeated CBCT images, which was contingent upon retrospective offline review comments from RTTs. These observations were made with reference to estimated bladder filling percentages or the presence of rectal gas as visualised on the CBCT scans. A second notable limitation is the potential for minor RTT errors when selecting specific AP-kV imaging parameters, as well as subjectivity in how RTTs reviewed and interpreted AP-kV images and made clinical decisions, influenced by their level of experience. This challenge was further compounded by the difficulty of conducting a visual volumetric comparison of bladder and rectal filling in real time with precise accuracy, comparing values from AP-kV images to those obtained from CBCT scans. A third limitation is the relatively small cohort size. It would have been beneficial to include a third modality for comparison, such as a bladder ultrasound scanner, to compare bladder volumes with those obtained from CBCT.

## Conclusion

This study supports the efficacy of daily AP-kV imaging prior to CBCT in patients undergoing prostate RT. This IGRT protocol enhances bladder filling and facilitates rectal gas emptying before each RT session. As a result, the DIM technique demonstrated marked superiority over the SIM technique in reducing CBCT dose and improving workflow efficiency, particularly in busy departments that do not use ultrasound bladder scanners. The DIM approach presented herein is characterised by its expeditious nature, and ease of integration into RT workflows, allowing for quick assessment of patient positioning and anatomy while the patient is in the actual treatment position.

## Declaration of competing interest

The authors declare that they have no known competing financial interests or personal relationships that could have appeared to influence the work reported in this paper.
